# CuInSe_2_ nanotube arrays for efficient solar energy conversion

**DOI:** 10.1038/s41598-019-53228-9

**Published:** 2019-11-14

**Authors:** Wipula Priya Rasika Liyanage, Manashi Nath

**Affiliations:** Department of Chemistry, Missouri University of Science and Technology, Rolla, MO 65409 USA

**Keywords:** Solar cells, Synthesis and processing

## Abstract

Highly uniform and vertically aligned *p-*type CuInSe_2_ (CISe) nanotube arrays were fabricated through a unique protocol, incorporating confined electrodeposition on lithographically patterned nanoelectrodes. This protocol can be readily adapted to fabricate nanotube arrays of other photoabsorber and functional materials with precisely controllable design parameters. Ternary CISe nanotube arrays were electrodeposited congruently from a single electrolytic bath and the resulting nanotube arrays were studied through powder X-ray diffraction as well as elemental analysis which revealed compositional purity. Detailed photoelectrochemical (PEC) characterizations in a liquid junction cell were also carried out to investigate the photoconversion efficiency. It was observed that the tubular geometry had a strong influence on the photocurrent response and a 29.9% improvement of the photoconversion efficiency was observed with the nanotube array compared to a thin film geometry fabricated by the same process. More interestingly such enhancement in photoconversion efficiency was obtained when the electrode coverage with the nanotube arrays as photoactive material was only a fraction (~10%) of that for the thin film device. Apart from enhancement in photoconversion efficiency, this versatile technique provides ample opportunities to study novel photovoltaic materials and device design architectures where structural parameters play a key role such as resonant light trapping.

## Introduction

There has been a rapid increase of research on nanostructured solar cells over the past few years owing to their promising potential of increasing photoconversion efficiency^1–3^. In this regard, nanoparticles have been widely explored as potential candidates for nanostructured photovoltaic (PV) solar cells due to their high surface area^1–4^. However, inefficient light scattering ability due to the small size of the nanostructured photoabsorbers (10–30 nm) and increased charge recombination due to electron scattering at particle boundaries has limited their efficiency improvement^5–7^. On the other hand, solar cells having high aspect ratio architectures of the photoabsorbers such as nanorods and nanowires have been reported to possess inherent advantages over conventional thin film devices^8–12^. The ability to fabricate solar cells with a larger tolerance of lattice-mismatched materials^13,14^ and the intrinsic strain relaxation property along with the greater absorption cross section make nanowires prospective candidates for the fabrication of low-cost and highly efficient solar cells^15^. In addition, well aligned nanowires have shown outstanding charge transport properties in solar cell applications^16–21^. Likewise, porous geometry consisting of periodically arranged nanoholes has also attracted significant attention as a surface modification technique of solar cells since these types of architectures have demonstrated efficient light trapping^22–25^ leading to improved photoconversion efficiency. Such improvements have been numerically simulated and experimentally demonstrated by several groups^26–29^. Therefore, a photovoltaic device that can combine high aspect ratio nanostructure along with the porous nanohole geometry can expectedly lead to enhanced efficiency by combining the advantages of both of those architectures. A well oriented array of photoabsorber nanotubes can be considered to be a combined architecture of nanohole and nanowire arrays. However, one should be able fabricate such well-ordered vertically oriented nanotube arrays systematically with uniform composition as well as, well-controlled physical parameters such as nanotube wall thickness, diameter, length and distribution pattern, to investigate the changes of photoconversion efficiency as a function of variation of structural parameters in order to optimize efficiency. Nevertheless, there are only a few reports available for such fabrication methods for vertically oriented nanostructure arrays which often involve the assistance of a hard template such as anodized aluminum oxide (AAO) or ZnO. Removal of such hard templates for exposing full functionality of the nanostructure arrays requires chemical treatment under harsh conditions such as highly acidic or basic solutions that can be also detrimental to the fabricated photovoltaic semiconductor’s performance. Moreover, using such rigid templates for nanotube growth also hinders the possibility of integrating these functional components into flexible modules that is becoming one of the prime areas of technological advances. It must be noted here that although nanowire arrays using AAO membranes^30–33^ and few nanotube arrays have been reported in the literature previously^34–37^, there are no reports of simple procedures for nanotube fabrication with pre-determined physical parameters.

In the present study, we have reported a facile route for the direct fabrication of highly oriented CuInSe_2_ nanotube arrays to investigate the advantage of both aspect ratio and nanohole architecture on the photoconversion efficiency. Our fabrication technique involving confined electrodeposition on lithographically patterned nanoelectrodes, allows us to precisely control structural parameters such as tube length, diameter, tube wall thickness, array distribution density, and periodicity which can then be optimized for achieving maximum efficiency. To the best of our knowledge, this is the first report on fabrication of CuInSe_2_ nanotube arrays. This simple few-step protocol has high flexibility for optimizing structure parameters for customized fabrication. Additionally, this protocol for direct growth of nanotube arrays on electrode surface eliminates the need of template removal using highly acidic or basic conditions thus making this process highly scalable and sustainable. The results obtained from photoelectrochemical characterization of the CuInSe_2_ nanotube arrays in this report has been compared with a thin-film photoabsorber morphology fabricated by similar process, which shows that the nanotube arrays exhibit significantly higher photoconversion efficiency compared to the film-like geometry. The protocol reported here is independent of the composition of deposition and can be applied to any photoabsorber, thereby increasing versatility of the process. Moreover, the ability to create nanotube architecture of complex ternary compositions from a single electrolytic bath, will have high significance in solar energy research.

## Experimental Section

### Growth of nanotubes

The CISe nanotubes were grown on indium tin oxide (ITO) coated conducting glass substrates by electrodeposition technique using an Iviumstat potentiostat following a protocol recently developed by the authors and referred to as confined electrodeposition on lithographically patterned nanoelectrodes (see ref. ^38^ for detailed process). In this protocol, first the desired array of nanoelectrodes with specific size and shape were patterned *via* electron beam lithography using Raith eLINE Plus nanolithography system on a polymethylmethacrylate (PMMA) coated substrate. The patterns were exposed on the surface of ITO-glass after developing the PMMA-coated substrate following *e-*beam exposure. An electrolytic bath containing Cu, In and Se precursors was prepared according to a reported procedure for CuInSe_2_ thin film deposition^39^ along with some modifications. A typical deposition bath consisted of 0.5 mM CuCl_2_, 2.0 mM SeO_2_ and 6.0 mM InCl_3_ with 0.1 M KCl as the supporting electrolyte in deionized water. After the solution was prepared 0.5 M HCl was added to adjust the solution pH to 2. The electrodeposition set-up consisted of a conventional three electrode system comprising lithographically patterned ITO-glass substrate as the working electrode, a platinum mesh as the counter electrode and Ag|AgCl electrode as the reference. Electrodes were vertically dipped in the electrolytic bath and electrodeposition was carried out under constant potential chronoamperometric conditions in stirred solution at room temperature for 20 s. In our experimental conditions, an applied potential of −0.7 V vs Ag|AgCl produced the optimal composition. After electrodeposition, the nanotube arrays were thoroughly washed with deionized water to remove any residues from the precursor solution and dried under a stream of nitrogen gas.

A layer of CdS has been widely used as the n-type layer of thin film CuInSe_2_ solar cells^40–44^ to create a p-n junction to effectively separate photo-generated electron hole pairs and is typically fabricated by chemical bath deposition (CBD). A uniform layer of CdS was thus obtained under well-controlled CBD conditions using a reported procedure^45^. The CBD process was carried out at 60 °C for 20 min to achieve a ~80 nm thick uniform layer. Low temperature or shorter time for CBD resulted in CdS films of low thickness. CISe photoabsorber arrays having a p-n junction were obtained by growing these nanotube arrays on nanoelectrodes patterned on CdS coated ITO glass.

### Characterization of nanotubes

Morphology and composition analysis of the nanotube arrays were carried out by using Helios Nanolab-600 scanning electron microscope (SEM) equipped with Energy Dispersive Analysis by X-rays (EDAX) detector (Oxford instruments) for elemental analysis. High resolution transmission electron microscopy (HRTEM) images and selected area electron diffraction (SAED) patterns were obtained using Tecnai F20 Transmission electron microscope. Powder X-ray diffraction (pxrd) patterns were collected using PANalytical’s X’Pert PRO Materials Research Diffractometer (MRD) employing Cu Kα radiation (λ = 1.5418 °A) at grazing angle incidence for the analysis of the crystal structure. Optical properties were studied with Cary 5 UV-Vis-NIR spectrophotometer. Photoelectrochemical (PEC) characterization, impedance and Mott-Schottky measurements of the nanotube arrays were performed using the IviumStat potentiostat and the light irradiation was provided with a 400 W Xe lamp with a light intensity of 100 mW/cm^2^. The electrolyte for photoelectrochemical measurement consisted of 0.1 M aqueous solutions of sodium sulfite, sodium sulfate, sodium acetate and the solution pH was adjusted to 4.6 with acetic acid^46^. Measurements were taken with the three electrode configuration as explained earlier using nanotube array as the working electrode.

## Results and Discussion

### Composition, morphology and crystal structure

In general, overall composition of the electrodeposited CISe depends on relative concentrations of the precursor ions in the deposition bath and applied potential. It has been reported previously that the deposition rates of Cu, In and Se varies with deposition time and applied potential^47^. Furthermore, it has also been reported that, under chronoamperometric conditions, during the first few minutes of deposition, there is a decrease in the In content while selenium content was increased in the deposited film before achieving a constant composition. On the other hand, when the applied potential for electrodeposition was increased the opposite trend was observed^47^. However, it has also been shown that high quality CISe devices can be fabricated with a range of compositions around the stoichiometric point^48^. Nevertheless, in our experiments, the observed composition changes in the deposition were minimum (less than 3%) since the deposition time was much less (~60 s). Typically, to observe noticeable changes in composition of thin films, longer deposition times are needed^39,47^. The deposition bath was also stirred very well do avoid limitations due to diffusion. Shorter deposition time and constant stirring also helped to avoid local pH variations near the working electrode and hence, avoid evolution of gaseous hydrogen and formation of indium hydroxide on the working electrode. Therefore, complexing agents such as citrates to maintain constant composition of the deposition and pH buffers to avoid pH variations were not required during the deposition process. In addition, at pH 2.0, the 0.1 M KCl added to the deposition bath acted as a background electrolyte to increase conductivity of the bath and also low pH helped prevent the formation of insoluble species such as indium hydroxide in the electrolytic bath.

For creating the nanostructure arrays, nanoelectrode patterns were created through lithography as described above on PMMA layer, which on development and removal of exposed resist, revealed the conducting substrate below the polymer. It can be assumed that during electrodeposition, the growth process is very similar to the typical thin film growth process as described above yielding similar composition. The confinement created through the PMMA layer is a physical barrier to maintain the shape and guide the direction of the deposit. During electrodeposition of nanotubes and thin films, we observed similar behavior  of the composition of the deposit as reported in previous literature by other researchers,^39,47^ in other words, at low applied potentials (~−0.5 V) indium content of the deposit was very low and it gradually increased with the increase of applied potential. The Cu and Se content of the deposit could be controlled by varying the concentration of the particular precursor ions in the solution to achieve desired composition. Most importantly, in our samples, there was no difference in the composition between thin film like geometry and the nanotube arrays when the deposition time was similar. Therefore, we assume that the confined environment has no significant effect to the typical growth process of CuInSe_2_ *viz* initially, the Cu^2+^ and Se^4+^ ions react to form CuSe through Se reduction and co-precipitation. CuSe further disproportionates to Cu_2_Se and Se^2−^. Cu_2_Se and Se^2−^ react with the In^3+^ from the solution leading to co-precipitation of CuInSe_2_^39^.

**Figure 1:**
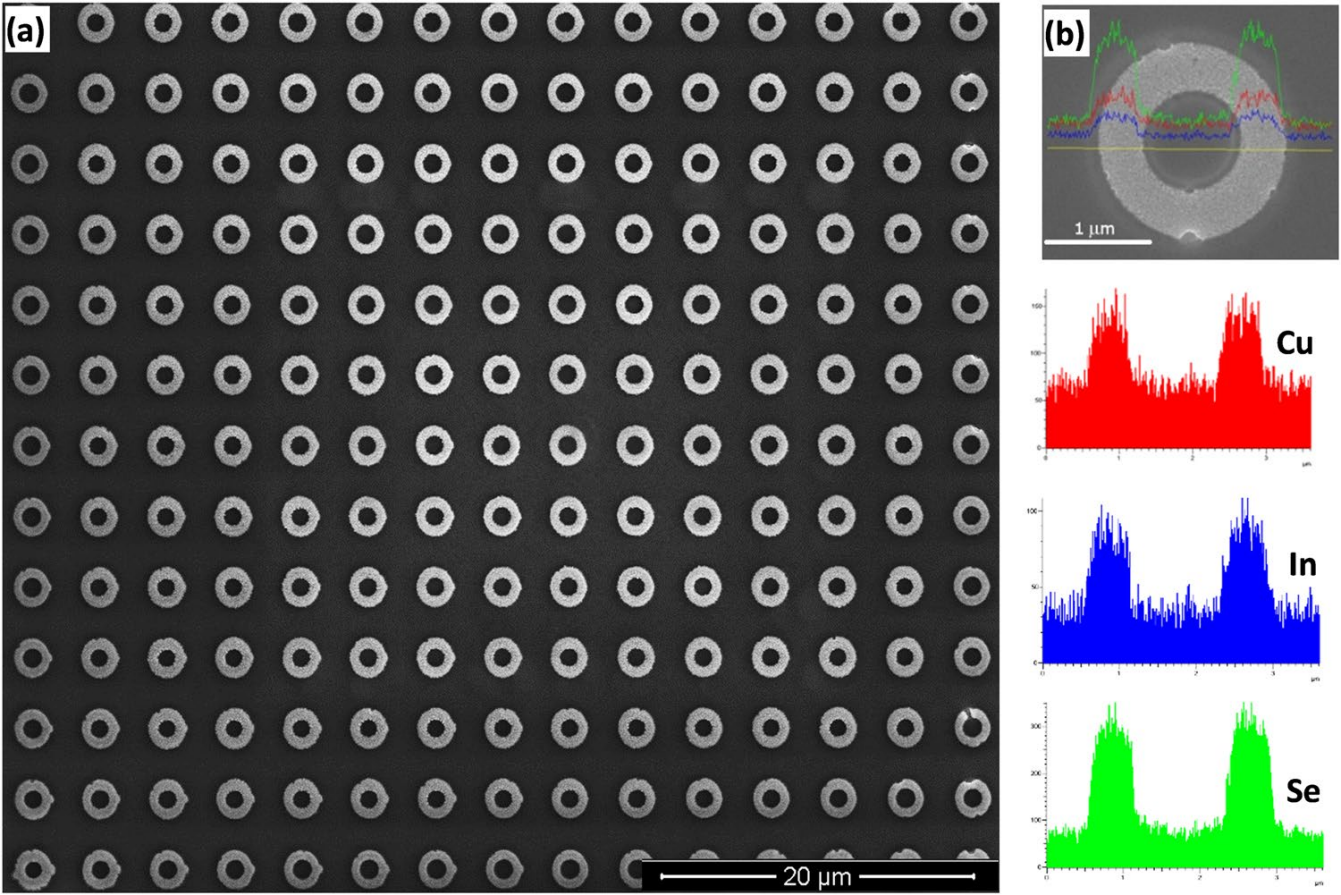
SEM image of (**a**) view from top of an array of fabricated CISe nanotubes. (**b**) EDAX line scan across a single nanotube indicating the Cu, In and Se elements.

An SEM image of as-deposited nanotube arrays has been presented in Fig. 1 showing the formation of uniform nanotubes exclusively on the patterned nanoelectrodes without any lateral growth or growth between the nanoelectrodes. The polymer coverage adjacent to the patterned nanoelectrodes confines the formation of the nanotubes during electrodeposition of the CISe inside the columnar channels and perpendicular to the substrate thereby leading to the formation of a highly oriented, vertical array of uniform nanotubes.

**Figure 2:**
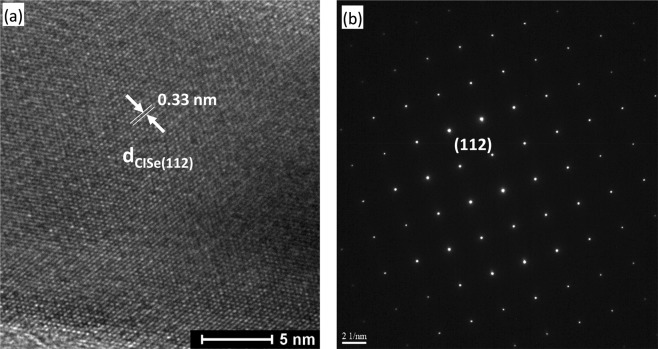
HRTEM image and SAED pattern showing dominant (112) crystal lattice fringes of CISe nanotube arrays.

The elemental analysis of as-deposited array by EDAX shows a near stoichiometric composition of CuInSe_2_ with a slight excess of Cu which also indicates that the deposit is *p-*type^48,49^. Figure S1(a) (supporting information) shows a tilted view of the pattern confirming the growth of these arrays up to the top surface of the polymeric resist and Fig. S1(b) (supporting information) shows a pattern that is intentionally damaged to better demonstrate the cross sectional view of tubular nature and the vertical alignment of the structure. Figure S2 (supporting information) shows a pattern that is covering a larger area and which indicates uniformity of the array in terms of size distribution of the individual nanotubes. The aspect ratio is another important parameter that governs the characteristics of the nanotube patterns and the device performance^15^. Figure S3 (supporting information) shows some typical examples illustrating the possibility of accurately controlling the diameter and wall thickness of the defined tube patterns created through this protocol. The length of the tube can be controlled by the thickness of the polymeric resist, which can be achieved by coating several layers of the polymer, while the tube diameter can be controlled by varying the pattern definition area. Hence this protocol has the unique advantage of fabricating customized nanotube arrays in a very cost effective manner. Authors have previously shown that the length of the tubes can also be grown beyond the thickness of the polymer by applying pulsed electrodeposition instead of continuous deposition^38^. Furthermore, it can be seen that due to the presence of polymer on the substrate, no CISe deposition takes place in other areas except on patterned nanoelectrodes. Therefore, structural parameters of fabricated nanotube arrays such as tube diameter, thickness of the nanotube walls, packing density as well as the distribution pattern of the array could be conveniently controlled by making desired adjustments to the pattern defined during the e-beam lithography process. This illustrates the novelty of this protocol to fabricate uniform nanotube arrays with pre-determined structure parameters. Some of the sample from the array was scraped off and loaded on a TEM grid for HRTEM analysis and selected area electron diffraction (SAED) pattern. As shown in Fig. 2, both HRTEM and SAED analysis reveals the highly crystalline nature of the nanotubes where the lattice fringes and diffraction spots could be indexed to the (112) lattice planes of CuInSe_2_ (JCPDS 35–1102).

**Figure 3:**
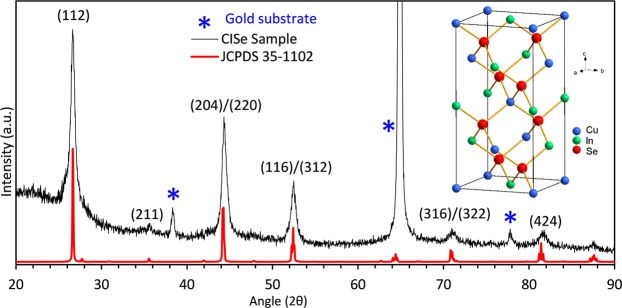
XRD pattern of CISe nanotube arrays matches well with the chalcopyrite structure of CISe (JCPDS 35–1102). The peaks labelled with an asterisk are from the gold substrate.

Crystal structure of the nanotube arrays was further examined by PXRD analysis. To obtain clean PXRD pattern, the CISe array was fabricated on Au coated glass substrates to avoid overlapping XRD peaks from the ITO background. Crystallinity of the deposition was improved upon annealing at 400 °C for 10 minutes in N_2_ atmosphere^50,51^. The crystallinity would improve further if more annealing was carried out but it also can lead to the loss of Se from the structure leading to compromising film quality and performance, therefore, additional annealing was not carried out. As shown in Fig. 3, the PXRD pattern matched with the standard chalcopyrite phase of CISe (JCPDS 35–1102). Other secondary phases such as Cu_2_Se, CuSe or In_2_Se_3_ or other impurity peaks were not observed in the PXRD pattern indicating superior phase purity of the tubular arrays. An average crystalline domain size of 20 nm was calculated from the peak broadening in the PXRD pattern by using the Scherrer equation^52^.

### Optical properties

**Figure 4:**
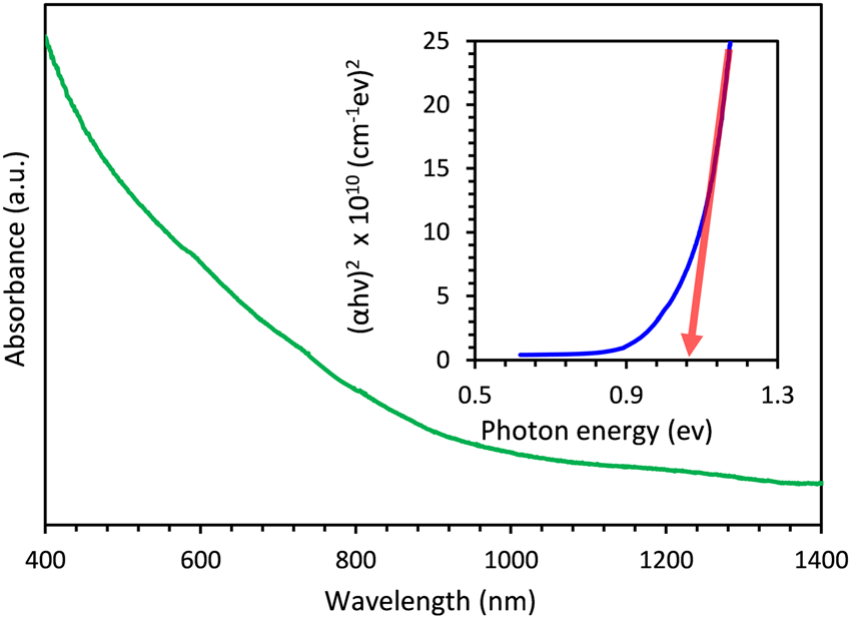
Absorption spectrum of the CISe nanotube array shows significant absorption over the visible region and inset shows the corresponding plot prepared for band gap determination considering variation of the absorption coefficient with photon energy.

Prepared nanotube arrays were examined using UV-Vis-NIR absorption spectroscopy to investigate optical properties. The spectrum as  shown in Fig. 4 shows a significant absorption in the visible region, which has been reported previously also as originating from the nonbonding copper 3*d* localized states^53^, suggesting suitability of the device for use in entire solar spectrum. Measured absorption coefficient values over the visible region are in the range of 10^4^ cm^−1^ and lies in the range of previously reported values for CISe^54–56^. The absorption curve has a characteristic tail in the long wavelength region which is typically observed in single crystalline and polycrystalline direct bandgap materials such as CISe. Such auxiliary absorption was well explained using photon assisted transition and Dow-Redfield model referring to the electric fields emerging from grain boundaries in polycrystalline materials^57^. Structural defects in grains and grain boundaries can also lead to gap states inside the band edges which show transitions in longer wavelength regions. When CISe samples are substantially heat treated at high temperature, it is known that loss of selenium occurs leading to selenium vacancies in the structure. In such cases, an additional transition has been observed in the low energy absorption region^58^. However, this sub band response was not observed from our nanotube device indicating that annealing did not lead to Se loss from these tubular arrays.

Optical bandgap, E_g_ for the CISe nanotube arrays, was estimated by considering the variation of absorption coefficient, *α*, with the photon energy, *hν*, using the plot of (*αhν*)^2^ vs *hν* according to the classical relation (*αhν*)^2^ = *A(hν-E*_*g*_) where *A* is a constant and the other symbols have their usual meaning.^54^ Linear region of the plot near the absorption edge was extrapolated to (*αhν*)^2^ = 0 to obtain the optical band gap energy as indicated in the inset of Fig. 3. The band gap of the nanotube arrays was determined to be 1.04 eV which corroborates very well with the observed CISe band gap energies at room temperature as reported in literature^54,59,60^.

### Photoelectrochemical (PEC) study of the nanotube array

A PEC analysis of the nanotube array was carried out in an acetate buffered electrolyte solution following a reported procedure^46^ where the redox couple in the solution forms a liquid junction with the top surface of the nanotubes. A typical electrochemical bath consisted of 0.1 M solutions of sodium acetate, sodium sulfite and sodium sulfate and the pH was adjusted to 4.6 with acetic acid. A conventional three electrode electrochemical set up with a platinum mesh as counter electrode, Ag/AgCl as the reference electrode and the fabricated nanotube array as working electrode was used in the experiment. The presence of polymeric e-beam resist covering space between the individual nanotubes in the array provides an added advantage by creating a barrier between the electrolyte and the back electrode. Otherwise, significant shunt conduction will be produced as a result of the contact of the electrolyte with the back electrical contact. The illumination for the nanodevice was provided with a Xenon lamp working in UVA zone (320–390 nm) with an intensity of 100 mW/cm^2^. The photochemical activity of the nanotube arrays was compared with a CISe thin film deposited over a fixed area on ITO-coated glass using similar procedure as described above.

**Figure 5:**
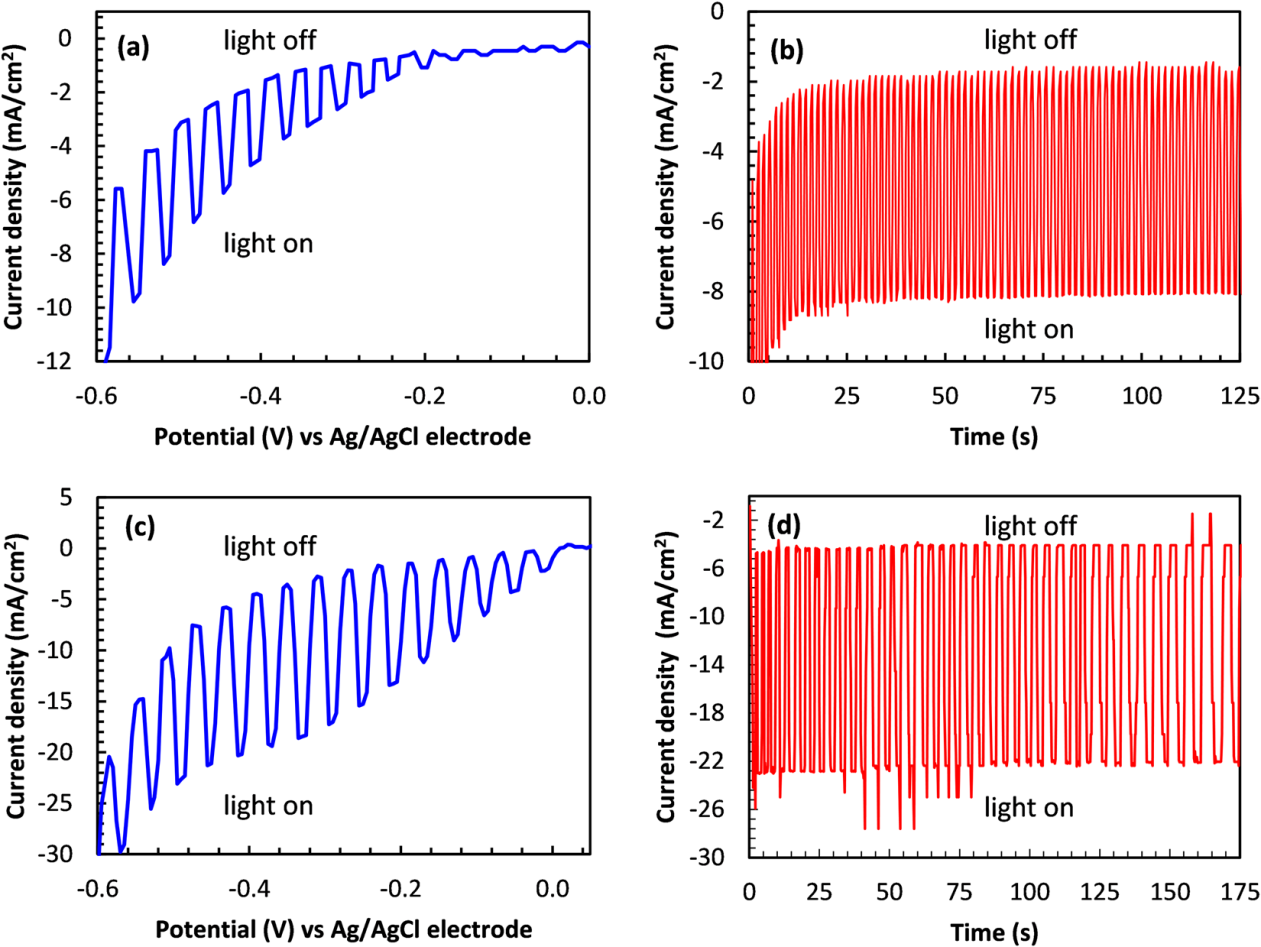
(**a**) Photoelectrochemical response of the thin film devices when the light source was turned on and turned off periodically under a potential sweep (**b**) Stability of the photo response of the thin film measured at an applied potential of −0.5V. (**c**) On- off response of the nanotube device under a potential sweep (**d**) Stability of the photocurrent of the nanotube device measured under applied potential of −0.5V.

Figure 5 shows the photocurrent response of the nanotube arrays compared with a thin film geometry of CISe fabricated by the same process. Current response of the devices was monitored while a potential scan was applied from + 0.3 V to −0.6 V vs Ag|AgCl at a scan rate of 5 mV/s.

The UV light source illuminating the devices were manually turned off and on periodically to record the dark current and the photocurrent responses, respectively. When the devices are illuminated a rapid increase of the cathodic current was observed and when the illumination was turned off, a rapid decrease of the current was observed confirming the generation of photocurrent in response to illumination. Typically for *p-*type materials, cathodic current is observed under illuminated conditions since the photogenerated electron transfer takes place from the conduction band of the semiconductor to the oxidant in the solution^61,62^. Usually the dark current is low when the photoelectrode is biased positive to the flat band potential (V_fb_) however, significant dark current flows at reduction potentials. The origin of electrons for increased dark current under continuous scan in the cathodic direction for p-type materials can be explained to be through valance band edge, and the mechanism by which they arrive at the surface has been correlated to the band bending at the electrochemical interphase under reduction conditions^61,63^. When the band bending occurs at reduction conditions, direct transfer of electrons across the space charge region from the valance band to a surface level in the band gap or to the edge of the conduction band may be possible leading to significant amount of dark current which alters the proper blocking behavior of the electrode. This can be minimized by introducing a suitable blocking layer to the photoabsorber. However, it is beyond the scope of this manuscript. The stability of the photocurrents were monitored by chronoamperometric method where the light source was turned on and off at constant intervals under a constant applied potential. Stable photocurrent could be obtained from the nanotube array for an extended period of time as shown in Fig. 5(d) and it also was noted that the fabricated nanotube arrays were stable in the electrochemical bath and did not undergo degradation under the experimental conditions. It is known that the acetate buffer solution can act as an efficient hole scavenger to prevent degradation of semiconducting material^46^.

**Figure 6:**
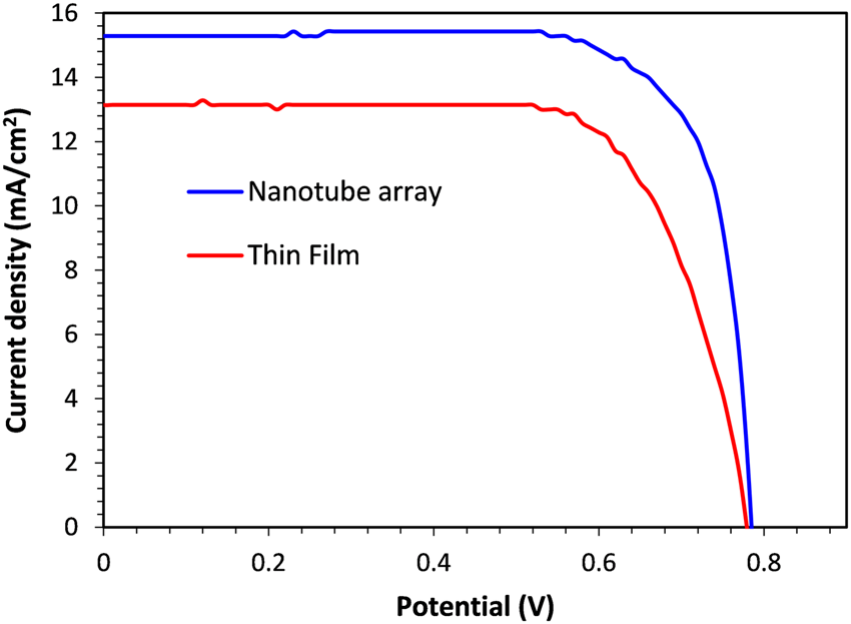
A Comparison of the photo current voltage (JV) performance of the CISe nanotube array and thin film devices.

Further analysis was carried out with nanotube arrays to understand photovoltaic behavior by fabricating PEC solar cell devices to measure photo conversion efficiency (η) taking into consideration the short circuit current density (J_SC_), open circuit voltage (V_OC_) and fill factor (FF). A heterojunction was made to the device by coating an *n-type* CdS buffer layer (~80 nm) on ITO glass by CBD method before fabricating nanotube arrays. The surface and crystallinity of CdS is of key importance to the performance of the solar cell device to improve separation of photogenerated electron-hole pairs and suppress recombination. Well controlled CBD leads to formation of a very uniform, crystalline layer of CdS semiconductor on the substrate. Authors have previously reported this process^38^ and followed similar procedure to grow the CdS layer reported in this study. As shown in the current density - voltage (*J*-*V*) plot given in Fig. 6 and summarized data given in Table 1, the cell fabricated with nanotube arrays could achieve a higher photo-conversion efficiency (8.85%) compared to a thin film solar cell device (6.82%) fabricated by the same procedure. It can also be observed that even though the *V*_*OC*_ is very close to each other for the two types of solar cell geometries, when morphology is changed to a tubular architecture, there is a notable enhancement of the *J*_*SC*_ and the *FF* for the nanotube array solar cell achieving ~30% increase in photo conversion efficiency compared to the thin film device.

**Table 1:** Summary of PV performance and impedance parameters of the CISe devices.

CISe geometry	*J*_sc_/mAcm^−2^	*V*_oc_/V	FF	η%	R_s_/ohm	R_ct_/kohm	CPE/Fcm^−2^
Nanotube array	15.28	0.785	75.78	8.86	11.69	1.22	7.1 × 10^–3^
Thin film	13.14	0.779	67.26	6.82	15.95	2.84	5.5 × 10^–5^

It should be noted that in addition to the improved performance, the nanotube array device has much less coverage (~10%) of the photo-active material on the electrode surface area compared to thin film device which covers the entire electrode surface area. The enhanced performance is likely due to the shortened carrier transport length coupled with better light scattering ability and enhanced light trapping of the nanotube array geometry which improve effective photoabsorption^64^. Similar observations have been previously reported for InP nanowire arrays where, 83% of the *J*_sc_ obtained from a thin film device was achieved by nanowire arrays despite material coverage of ~12%^65^. In addition, it has been demonstrated that CdSe nanowire arrays shows higher minority carrier collection efficiency and has the ability to absorb low energy photons more efficiently than thin film electrodes fabricated to the similar thickness^66^.

Enhancement of the fill factor in the nanotube array device can be related to the enhanced charge transport across the junction which often produce a competition between minority carrier collection across the junction and the surface recombination^63^. The combination of one-electron transfer redox couple with the increased junction area due to the tubular architecture of the device therefore, improves charge transfer across the junction compared to the surface recombination because, at a given illumination intensity, the minority carrier flux density decreases as the surface area of the junction increases. This can lead to an enhanced fill factor in the nanotube device compared to the thin film device as observed in this article. The nanotube array devices reported here provides proof of concept that CISe nanotube arrays can be fabricated by this method and shows enhanced photoconversion under illumination. The significantly improved photoconversion efficiency shown by the CISe nanotube array indicates that this tubular morphology can be further tuned to increase photoconversion efficiency by manipulating physical parameters such as nanotube pore diameter, wall thickness, and distribution pattern of the array to further improve light scattering, photoabsorption and light trapping with the aid of some simulation studies since this fabrication protocol has the capability to make arrays with pre-determined parameters.

**Figure 7:**
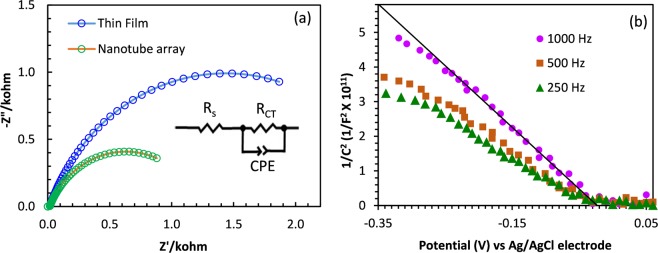
(**a**) Nyquist plots of nanotube array and the thin film device with the corresponding equivalent circuit as the inset. (**b**) Mott-shottky plots of the CISe nanotube array in the dark. Data was recorded at 1000 Hz, 500 Hz and 250 Hz and the line was drawn considering the linear region of the 1000 Hz data.

Electrochemical Impedance Spectroscopy (EIS) analysis was carried out with the nanotube device which showed a typical Nyquist plot as shown in Fig. 7(a), and the charge transfer resistance (*R*_*ct*_), solution resistance (*R*_*s*_) and constant phase element (*CPE*) indicating the double layer capacitance was determined by fitting the measured impedance data in to an equivalent circuit model. Evaluated EIS parameters have been summarized in Table 1. The nanotube array showed a much less charge transfer resistance and higher double layer capacitance than that of thin film geometry which can in turn leads to a higher current density shown by the nanotube array due to the efficient carrier generation and transport provided by the tubular geometry.

The electrodeposited CISe was further characterized through Mott-Schottky analysis  to evaluate the film quality. Figure 7(b) shows the Mott-Schottky plot calculated from the EIS data by considering the capacitance, *C*, vs applied potential at 1000 Hz, 500 Hz and 250 Hz frequencies with a small AC amplitude (10 mV) at each potential^62^. The analysis was carried out in the dark and the negative slope of the linear fit confirms that electrodeposited CISe are *p-*type conductors supporting the observed cathodic photocurrent in photoelectrochemical measurements. The flat band potential was obtained from the intercept of the slope with the x-axis from the line drawn through the linear region of the 1000 Hz data. The flat band potential was estimated to be −0.03 V (vs Ag|AgCl electrode), which is in good agreement with the observed photocurrent onset potentials in the photoelectrochemical measurements. Generally, there is an increase in the resistive component of the electrode with applied potential in the dark due to the formation of a depletion layer consisting of immobile charges in the double layer region. Since free carriers are formed under illumination, this resistance is eliminated. Therefore, the position of the flat band can be considered as the point where onset of the photocurrent starts eliminating this resistance^61^.

## Conclusions

We demonstrated a simple protocol for the direct fabrication of CISe nanotube arrays on a conducting substrate with controllable design parameters without using hard templates such as AAO or other sacrificial templates. Use of e-beam lithography as the patterning process enables one to utilize the potential of this ever-growing powerful technique to manipulate all the key design parameters of the nanotube array such as pore diameter, wall thickness, distribution pattern, distribution density, etc., for optimizing the photo absorption and maximizing the photoconversion efficiency to obtain highly efficient solar cell devices. The e-beam resist used in the process provides a soft and flexible matrix for the growth of vertically aligned nanotube arrays and the thickness of the resist can be used to control the length of the nanotubes. The photoelectrochemical measurements showed that these nanotube arrays are capable of producing higher current densities despite the use of much less active material coverage compared to thin film devices making it is possible to use expensive materials for photo conversion at a lower cost. The strong influence for the photoconversion by nanotube arrays can be caused by the elongated effective absorption length, multiple light scattering by tubular architecture of the array, and short carrier transport distance. In general, we believe the present work will have large implication for solar energy research and will initiate further studies. Such patterned growth of high quality nanostructures would be very useful in miniaturizing nanodevices as well as fabricating highly sensitive photodetectors and various other optoelectronic devices. Further improvements to the PV performance can be made by the application of surface passivation layers and optimizing the thickness of the buffer layer to minimize recombination losses. Another avenue for improvement is the introduction of Ga to the system so that the bandgap can be manipulated for a broader light harvesting range. The versatility of the protocol reported here also ensures the possibility of providing such structural variance in the photoabsorber layer. Once optimized for a given system, the same concept introduced by this paper has the potential to be transferred to other lithographic aided growth techniques such as UV-Lithography to grow the identified patterns in large scale.

## Supplementary information

Supplementary information for publication
