# Supplementary material for: CuInSe2 nanotube arrays for efficient solar energy conversion
Source: Sci Rep. 2019 Nov 14;9:16751. doi: 10.1038/s41598-019-53228-9 (PMC6856161; doi:10.1038/s41598-019-53228-9)
Supplement: Supplementary file 1 — Supplementary information for publication [file 41598_2019_53228_MOESM1_ESM.docx]

Supplementary Information

CuInSe_2_ nanotube arrays for efficient solar energy conversion

Wipula Priya Rasika Liyanage, Manashi Nath^*^

Department of Chemistry, Missouri University of Science and Technology, Rolla, MO 65409

*Corresponding author E-mail: nathm@mst.edu

Figure S1 SEM image of (a) a tilted view of a CISe nanotube array showing the growth of tubes to the top limit of the polymer layer. (b) an array of nanotubes was scratched and some polymer was removed to show the cross-sectional view of tubes. These tubes were grown to a length of 800nm.


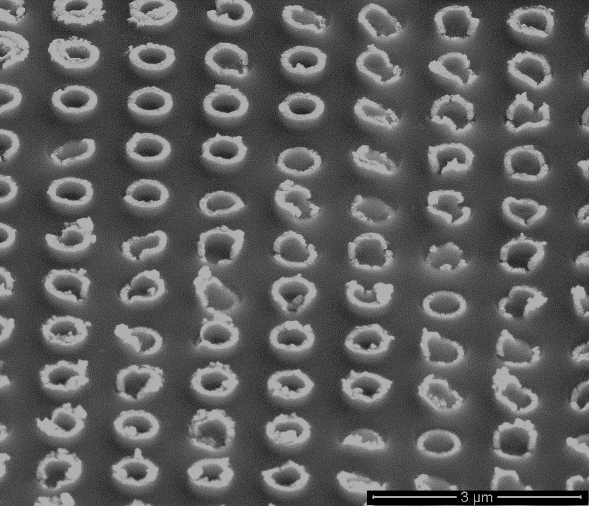

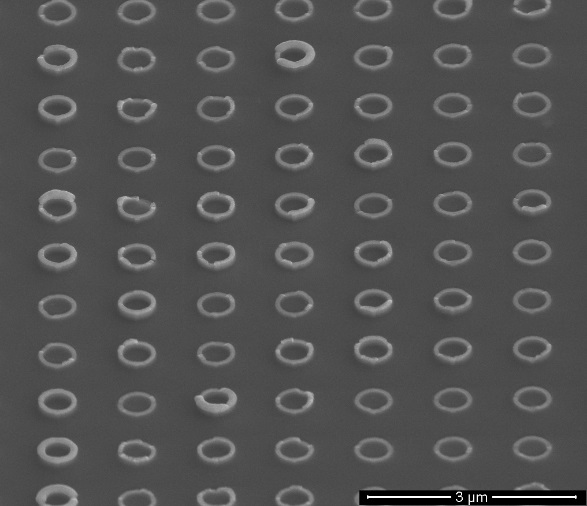


(a)

(b)


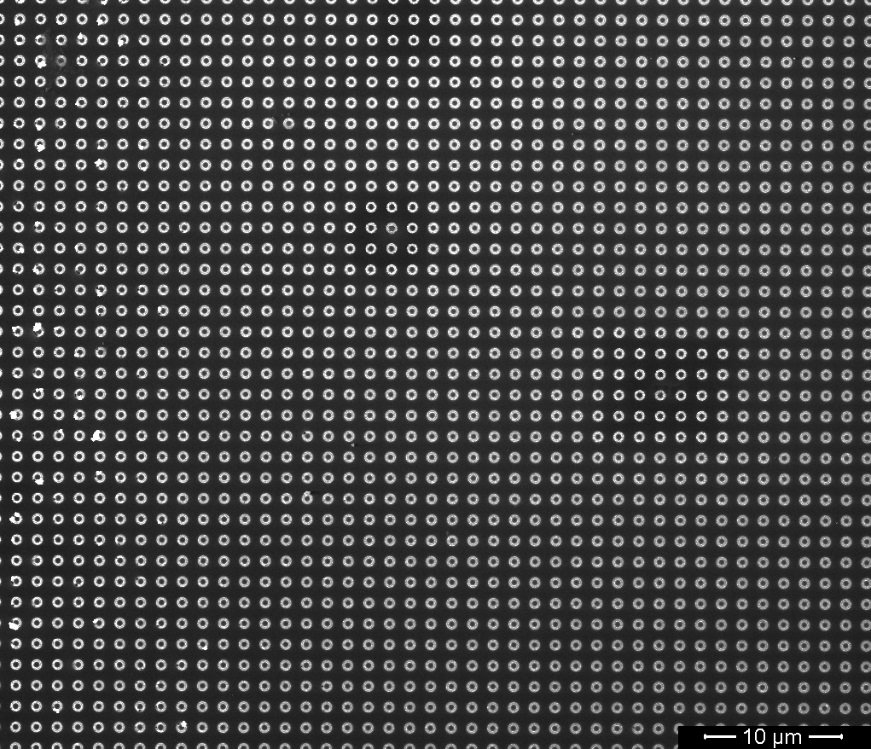


Figure S2 SEM image of a CISe nanotube array covering a larger area showing the uniformity of the array indicating the ability of this protocol to fabricate nanotube arrays with accurate structure parameters.

Figure S3 SEM image of (a) CISe nanotube arrays to show the possibility of accurately changing the diameter, wall thickness and distribution density of nanotubes created through this protocol. Also shown are arrays of tubes with controlled outer diameter and wall thickness illustrating versatility of this method: (b) 830nm and wall thickness of 250nm; (c) 780 nm and wall thickness of 175nm’ (d) 2.3um and wall thickness of 350nm. The length of the tubes are mainly controlled by the thickness of the polymer (~ 800nm in this study).


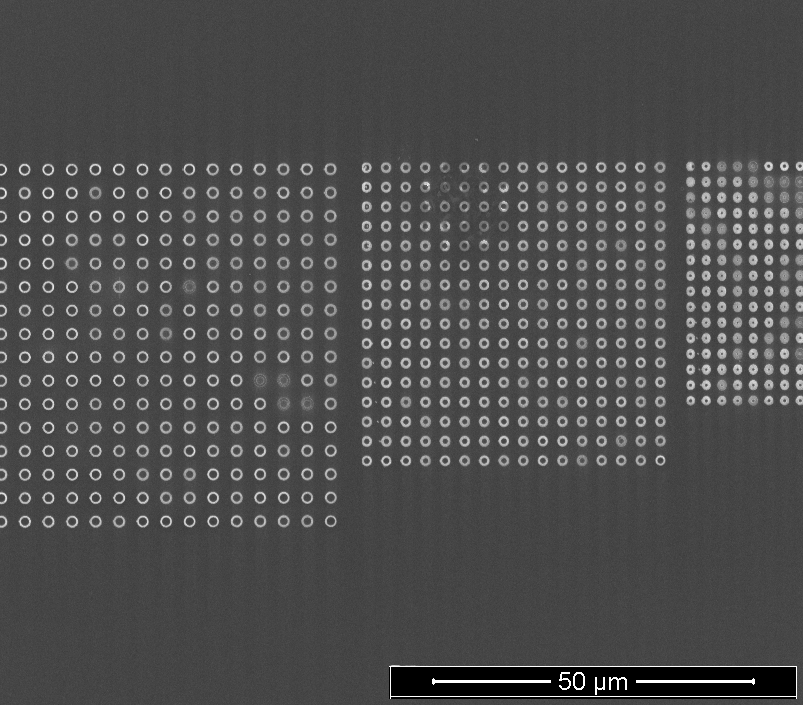

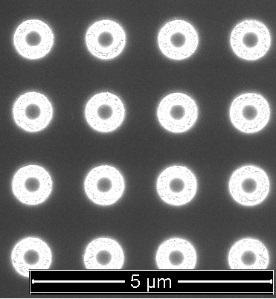

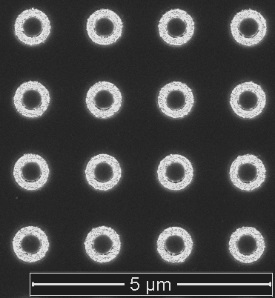

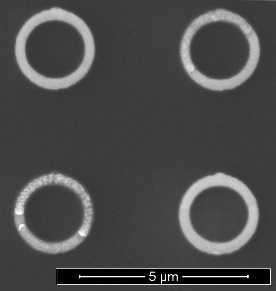


(a)

(b)

(c)

(d)
